# Differential Oral Microbiota and Serum Cytokine Signatures in Age-Grouped Patients with Marfan Syndrome

**DOI:** 10.3390/biomedicines13020330

**Published:** 2025-01-31

**Authors:** Erick Ricardo Ordaz-Robles, María Elena Soto, Paulina Hernández-Ruiz, Alma Reyna Escalona-Montaño, Luis Alejandro Constantino-Jonapa, Amedeo Amedei, María Magdalena Aguirre-García

**Affiliations:** 1 Unidad de Investigación UNAM-INC, División de Investigación, Facultad de Medicina UNAM, Instituto Nacional de Cardiología Ignacio Chávez, Mexico City 14080, Mexico; ricardoordaz3113@gmail.com (E.R.O.-R.); paulina.hdez@hotmail.com (P.H.-R.); almaescalona@comunidad.unam.mx (A.R.E.-M.); biologia0712@gmail.com (L.A.C.-J.); 2 Research Direction, Instituto Nacional de Cardiología Ignacio Chávez, Juan Badiano 1 Col. Sección XVI, México City 14080, Mexico; elena.soto@cardiologia.org.mx; 3 Cardiovascular Line, Hospital ABC Observatorio, Mexico City 01120, Mexico; 4 Department of Experimental and Clinical Medicine, University of Florence, 50134 Florence, Italy; 5 Network of Immunity in Infection, Malignancy and Autoimmunity (NIIMA), Universal Scientific Education and Research Network (USERN), 50139 Florence, Italy

**Keywords:** oral microbiota, Marfan syndrome, cardiovascular disease, cytokines

## Abstract

**Introduction:** Marfan syndrome (MFS) is an autosomal dominant genetic disorder, caused by a mutation in the FBN-1 gene, affecting the cardiovascular, musculoskeletal, ocular, and central nervous systems. Cardiovascular abnormalities associated with MFS lead to different pathological conditions, such as cardiac arrhythmias, coronary artery disease, and aortic dilatation. The latter are the primary causes of mortality in MFS patients. To date, the role of altered oral microbiota (OM) in MFS is unknown, and so the aim of our study was to determine whether there are differences in the oral microbiota of MFS patients with aortic dilatation and non-dilatation. **Methods:** We enrolled 36 MFS patients, who were divided into groups with aortic non-dilatation (n = 12) and with aortic dilatation (n = 24). Dental plaque samples were used for OM analysis, and serum was used for cytokine evaluation. **Results:** The main genera were compared between patients with aortic dilatation and non-dilatation, revealing three genera with significant differences: *Actinomyces* (*p* = 0.007) and *Rothia* (*p* = 0.002) were more abundant in those with aortic dilatation, while *Fusobacterium* (*p* = 0.044) was more abundant in non-dilatation patients. However, no significant differences in cytokine levels were observed between the presence and absence of aortic dilatation, except that the IL-1β levels were higher in non-dilatation patients (165.09 pg/mL) than in those with dilatation (117.15 pg/mL), with a significance of *p* = 0.057. **Conclusions:** This study represents the initial, tentative pilot study to understand the relationship between oral health and systemic conditions in patients with Marfan syndrome.

## 1. Introduction

Marfan syndrome (MFS) is an autosomal dominant genetic disorder caused by a mutation in the FBN-1 gene, which encodes the protein fibrillin-1, leading to connective tissue abnormalities. It is estimated that the incidence of MFS is approximately 1 in every 5000 individuals, regardless of ethnic group and/or geographical region. Due to its rarity, diagnosing MFS is challenging, and currently, the syndrome’s prevalence in Mexico remains undefined [1,2,3].

This disease affects the cardiovascular, musculoskeletal, ocular, and central nervous systems. However, the condition and damage severity vary from one patient to another, with even the signs and symptoms differing among multiple cases within the same family [4].

Cardiovascular abnormalities associated MFS lead to a variety of pathological conditions, such as cardiac arrhythmias, coronary artery disease, left ventricular hypertrophy, congestive heart failure, and aortic dilatation and dissection. The latter two are the primary causes of mortality in MFS patients [5].

According to the Brussels Convention of the Ghent Nosology [6], there are five criteria for diagnosing MFS; at least three must be present for a positive diagnosis. These criteria include clinical characteristics such as an FBN1 gene mutation; ectopia lentis; abnormalities in the aortic root; a family history of the syndrome; and a systemic score of ≥7, which also incorporates musculoskeletal alterations. Notably, the symptoms of MFS can manifest at various stages of life, making early diagnosis particularly difficult [7].

In the dental field, MFS presents characteristic signs, most notably a high palatal vault and a bifid uvula, as well as other oral manifestations, including long, narrow teeth; malocclusions; mandibular prognathism; temporomandibular joint disorders; and structural changes in the uvula [8]. These maxillary and mandibular alterations can lead to significant changes in tooth eruption and organization, a condition known as dental crowding. As a result, there is more food accumulation, which, in turn, fosters plaque buildup that increases the risk of pathogenic microorganism proliferation, elevating the likelihood of developing caries and periodontal disease [9].

The oral cavity hosts one of the most clinically relevant microbiological niches, as it is one of the primary entry points for microorganisms into the body, with more than 700 different species of bacteria. This niche represents the second-largest and most diverse microbial community after the intestine; however, the oral microbiota is much less studied.

The oral microbiota’s composition depends on various factors such as geographic region, ethnicity, and age, and it is well documented that infectious diseases, such as dental caries, are linked to the development of systemic conditions, including metabolic diseases, cardiovascular diseases, and cancer [10,11,12,13].

During the stages of dental caries development and across the two types of dentition (primary and permanent), the predominant bacteria vary. For example, the dominant bacteria in the initial demineralization of both primary and permanent teeth are *S. salivarius* and *Streptococcus parasanguis*. Meanwhile, the bacteria most commonly detected in deeper dental cavities in the primary dentition include *S. salivarius, S. parasanguis,* and *Corynebacterium*, while in the permanent dentition, *Actinomyces gerencseriae, S. salivarius, S. parasanguis, Campylobacter,* and *Selenomonas* are most common [11].

There is known to be a connection between the OM and cardiovascular diseases, which include conditions such as congestive heart failure, cardiac arrhythmias, valvular diseases, strokes, and coronary artery diseases (including atherosclerosis and acute myocardial infarction). These conditions are associated with the presence of microorganisms such as *Streptococcus, Veillonella, P. gingivalis, F. nucleatum, T. forsythia,* and *Neisseria*, which are derived from OM [10,11,12].

Studies have reported that when OM microorganisms translocate into the bloodstream, they promote low-grade systemic inflammation, triggering a response mediated by Th (T helper) cells and increasing the levels of TNF (Tumor Necrosis Factor), IL (Interleukin)-1β, IL-6, and IL-8 [14,15,16].

Due to the chronic low-grade inflammation associated with OM dysbiosis, it should be considered a critical factor in the development of cardiovascular alterations in MFS patients.

A relevant point to highlight is that in most patients with Marfan syndrome (MFS), the palatal vault is deep, and they have long, narrow teeth, often accompanied by malocclusion associated with mandibular prognathism or retrognathia. These anatomical conditions favor the accumulation of dental plaque on the tooth surfaces, significantly increasing the risk of cavities and periodontal diseases. This is especially relevant since the most severe complications in MFS are related to aortic dilatation or dissection, valvular heart failure, myocardial infarctions, and other cardiovascular diseases.

In the case of patients with MFS who have received frequent professional dental cleanings, there is a lower incidence of cardiovascular diseases, suggesting that maintaining proper oral hygiene helps improve their overall health [17]. However, there are no studies on the relationship between the microbiome and clinical status or whether dental and maxillary deformities further affect cardiovascular condition (R1A1).

The aim of this study was to determine whether there are differences in the oral microbiota in Marfan syndrome patients with aortic dilatation and non-dilatation.

Therefore, it could be proposed as an additional factor in the treatment of this disease.

## 2. Materials and Methods

### 2.1. Patients

This project was approved by the ethics and scientific committees of the Instituto Nacional de Cardiología “Ignacio Chávez”, with protocol number PT-18-079, Ref. INCAR-DG-DI-90-2018, and approved on 6 June 2018. An observational, cross-sectional study was conducted on a cohort of patients recruited between August 2018 and January 2020. The study population included patients of all genders and ages who attended the Instituto Nacional de Cardiología “Ignacio Chávez”. Patients were diagnosed according to the Ghent criteria (family history, aortic dilatation, ectopia lentis, or a systemic score ≥7), and those who met two or more of these criteria were selected.

Patients were excluded if they had missing teeth, preventing the collection of dental plaque samples, or if they engaged in chronic alcohol consumption, smoking habits, or drug use. Other exclusion criteria included patients who had received antibiotics within the last month prior to sampling, as well as pregnant or breastfeeding women.

Patients were grouped according to age ranges. While there is no clear consensus on how to categorize age groups throughout the life cycle, most countries within the Economic Commission for Latin America and the Caribbean divide childhood into three stages: early childhood (0 to 5 years), middle childhood (6 to 12 years), and adolescence (13 to 18 years) [18]. In Mexico, CONAPO (National Population Council) classifies age groups as follows: early childhood (0 to 5 years), childhood (6 to 14 years), youth (15 to 24 years), young adults (25 to 44 years), mature adults (45 to 59 years), and older adults (60 years or more) [19].

Based on these classifications, this study proposes categorizing patients into three groups: child (5 to 13 years), teenager (14 to 19 years), and adult (20 to 60 years).

### 2.2. Oral Evaluation

A directed medical history and dental evaluation were conducted through a dental chart, which recorded parameters such as decayed, missing, and filled teeth. Additionally, the risk of caries was assessed and classified as very low, low, medium, high, or very high, based on the DMF-T index (decayed, missing, and filled teeth) [20]. The anatomical condition of certain oral structures was also documented, including the type of palate, the uvula shape, and the presence or absence of dental crowding. This evaluation was performed individually for each participant who chose to take part in the study.

### 2.3. Biological Samples

Peripheral blood samples were collected from patients using polyethylene terephthalate tubes (BD Vacutainer REF: 367863, REF: 368159, USA) containing EDTA for plasma and polymer gel for serum. The plasma and serum were separated by centrifugation and immediately stored at −80 °C for future analysis. Dental plaque (DP) samples were collected by scrapping the vestibular and lingual surfaces of all teeth. The collected samples were placed in Eppendorf tubes containing 70% ethanol and stored at −20 °C. These samples were immediately stored at −80 °C to preserve their integrity for subsequent analysis.

### 2.4. DNA Extraction and 16S rRNA Sequencing

DNA extraction from dental plaque samples was performed using the EZ-10 Spin Column Genomic DNA Minipreps kit, Animal (Bio Basic Inc., CAT#: BS427-50 Preps, Markham, ON, Canada), following the manufacturer’s instructions. The DNA concentration, purity, and integrity were verified using a spectrophotometer (Nanodrop 1000, ThermoFisher, Waltham, MA, USA) and gel electrophoresis (ChemiDoc Image System, BioRad, Hercules, CA, USA). The extracted DNA was sent to Novogene, where 16S libraries were prepared and sequenced using the NovaSeq 6000 PE250 platform (Illumina, San Diego, CA, USA). In particular, the V3–V4 hypervariable region was amplified using the primer pair 341F (CCTAYGGGRBGCASCAG) and 806R (GGACTACNNGGGTATCTAAT).

### 2.5. Bioinformatic Analysis

Demultiplexed raw FastQ files (R1 and R2) were processed using QIIME2, v.2020.11, USA [21]. The DADA2 plugin was employed to merge paired-end FastQ files, denoise the data by removing chimeras, and construct a table of operational taxonomic units (OTUs) [22]. Taxonomy assignment was conducted using the eHOMD database v.15.2 (https://www.homd.org/) at 99% identity, pre-trained for the V3-V4 region [23,24].

Rarefaction was performed at a sampling depth of 50,000 sequences per sample. Alpha diversity was estimated using the Shannon and Chao1 indexes, while beta diversity was assessed using Bray–Curtis dissimilarity and visualized through Principal Coordinate Analysis (PCoA). Permutational Multivariate Analysis of Variance (PERMANOVA, 999 permutations) was applied to test for significant differences between groups.

### 2.6. Cytokine Quantification

Serum cytokines were quantified using the LEGENDplex™ Human Essential Immune Response Panel kit, 13plex (Biolegend, CAT#: 740930, San Diego, CA, USA), including the following cytokines: IL-1β, IL-4, IL-6, IL-8, IL-10, TNF, IFN-γ, and TGF-β1. Samples were evaluated using a FACSAria flow cytometer (Biosciences, San Jose, CA, USA), and raw data were analyzed with the LEGENDplex™ Data Analysis Software Suite BioLegend 8999 BioLegend Way San Diego, CA 92121 The FlowVigene™ V10 (https://legendplex.qognit.com/workflow, e.g., accessed on 3 April 2024). Each assay was performed in triplicate.

### 2.7. Statistical Analysis

The data were analyzed using R software version 4.1.2. To compare taxonomic profiles between the two study groups and analyze the alpha diversity data, the Mann–Whitney U test was applied for the analysis of two independent samples, and the Kruskal–Wallis test was used for more than two independent samples.

For the clinical data, the Kruskal–Wallis test was used. All analyses were two-tailed, and a significance level of *p* ≤ 0.05 was set.

## 3. Results

### 3.1. Demographic Data

A total of 36 patients, both male and female, aged between 5 and 55 years, were included, all diagnosed with at least two of the Ghent criteria, with aortic dilatation being the second most frequently recorded criterion among these patients. The groups were classified according to age as follows: children (n = 15), teenagers (n = 10), and adults (n = 11). The demographic and clinical data of the patients are summarized in Table 1. It is relevant to highlight the systemic score section, which includes facial traits such as a high-arched palate and dental crowding. It was observed that 75% of the patients had a family history, and 66.7% of the total sample presented aortic dilatation. Among the oral characteristics, a deep palatal arch (80.6%) and bifid uvula (77.8%) had a high incidence rate. It should be noted that no statistical differences were found in the comparative analysis of the clinical features between the different groups.

### 3.2. Oral Microbiota Diversity

**Figure 1:**
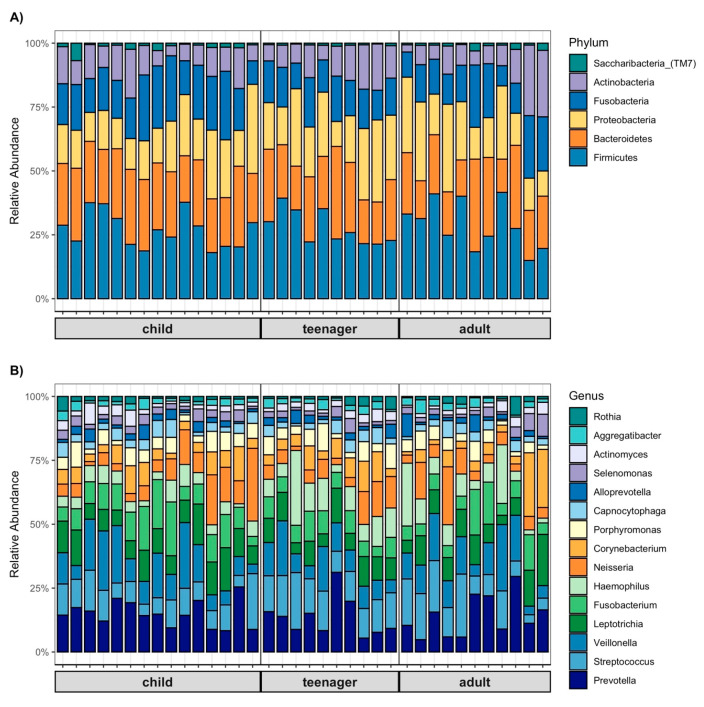
Relative abundance at the phylum level **A**) and genus level **B**) within the child (5–13 years), teenager (14–19 years), and adult (20–60 years) age groups. The identified phyla and genera are grouped in a proportion of >1%.

The OM analysis showed that the six most abundant phyla were *Firmicutes, Bacteroidetes, Proteobacteria, Fusobacteria, Actinobacteria, and Saccharibacteria (TM7)*, accounting for 98% of all taxa. When analyzed by age groups, the relative abundance of *Firmicutes* was 27% in the child group, with an increase in this phylum observed in both the teenager and adult groups, at 29%. On the other hand, *Bacteroidetes* presented a relative abundance of 25% in children, decreasing to 23% and 22% in the teenager and adult groups, respectively. *Proteobacteria* was the most abundant phylum in teenagers (22%) and accounted for 18% in children. *Fusobacteria* was the most abundant phylum in children, with a relative abundance of 19%, while its abundance was 15% in teenagers. Finally, *Actinobacteria* and *Saccharibacteria_ (TM7)* showed similar abundances of 10–11% and 2–1%, respectively (Figure 1A).

Regarding the distribution by gender, the 15 most abundant bacteria are shown (Figure 1B). *Prevotella* was the most abundant genus in all three age groups, with a relative abundance between 12–13%. *Streptococcus* was the most abundant genus in teenagers (12%) and the least abundant in children (9%). Meanwhile, *Veillonella* was the most abundant genus in both children and adults, with 11% in each group, and accounted for 9% in teenagers. Additionally, *Leptotrichia* showed a relative abundance of 9% in all three age groups. The comparison between groups revealed a statistically significant difference in the genus *Haemophilus* (*p* = 0.027) (Figure 1B).

**Figure 2:**
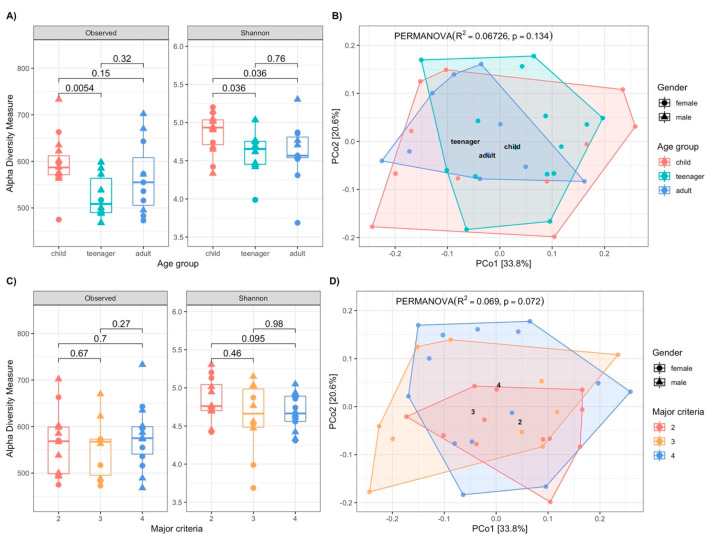
Alpha and beta diversity (PERMANOVA, R2, *p*-value) **A**) Analysis of alpha diversity according to age groups based on the Shannon index and the number of OTUs observed (*p*-value, Mann–Whitney). **B**) Principal coordinate analysis for beta diversity by the Bray–Curtis index according to age groups (R^2^ = 0.067, *p* = 0.134). **C**) Alpha diversity analysis based on the Shannon index and the number of OTUs observed according to the number of major criteria (Ghent) present in each subject (*p*-value, Mann-Whitney). **D**) Principal coordinate analysis for beta diversity by the Bray–Curtis index according to the number of major criteria (Ghent) present in each subject (R^2^= 0.069, *p* = 0.072, Mann–Whitney).

To evaluate the OM diversity, a significance analysis was performed using the α-diversity indices (observed OTUs and Shannon indices) grouped by age and by the number of major criteria. Regarding age groups, both indices showed greater diversity in the child group, with differences observed between the child and teenager groups (observed OTUs, *p* = 0.0054, Kruskal–Wallis; Shannon, *p* = 0.036, Kruskal–Wallis) (Figure 2A).

The beta diversity analysis using the Bray–Curtis index, shown in the principal coordinate plot by age group (Figure 2B), determined that 6.7% of the variance was related to this variable (R^2^ = 0.067, *p* = 0.134, PERMANOVA).

On the other hand, when the same patients were grouped according to the number of major Ghent criteria, the alpha diversity indices showed no differences between those meeting two, three, and four criteria (Figure 2C). In the beta diversity analysis, 6.9% of the variance was related to the categories of the Ghent criteria (R = 0.069, *p* = 0.072, PERMANOVA) (Figure 2D).

**Figure 3:**
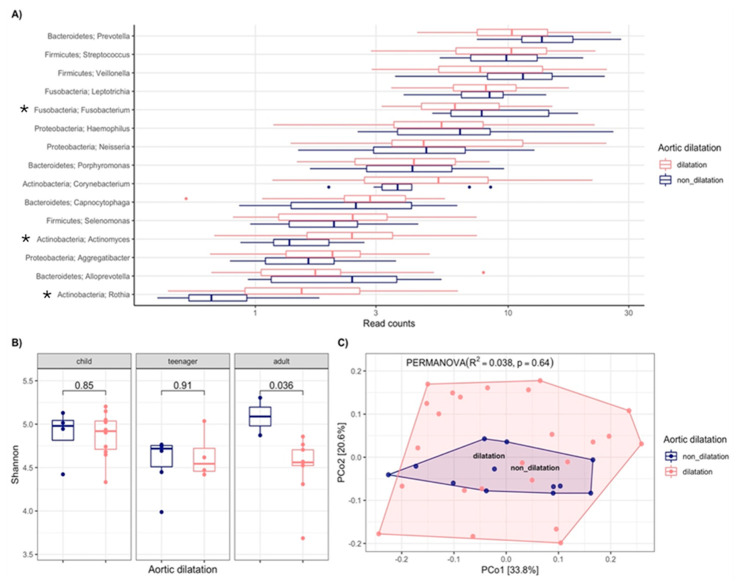
Analysis of oral microbiota in subjects with MFS according to aortic dilatation. **A**) Relative abundance of the main genera. **B**) Analysis of alpha diversity in relation to age groups and aortic dilatation based on the Shannon index. **C**) Principal coordinate analysis of beta diversity by the Bray–Curtis index in relation to aortic dilatation R^2^ = 0.038, *p* = 0.064. * Correspond to significant difference.

Since cardiovascular alterations are the leading cause of mortality in MFS patients, an OM analysis in relation to aortic dilatation was performed. The main genera were compared between patients with aortic dilatation and non-dilatation, showing three genera with significant differences: *Actinomyces* (*p* = 0.007) and *Rothia* (*p* = 0.002) were more abundant in those with aortic dilatation, while *Fusobacterium* (*p* = 0.044) was more abundant in non-dilatation patients (Figure 3A).

Alpha diversity analysis based on the Shannon index was conducted to determine the microbiota abundance across the three different age groups in relation to dilatation, showing a significant difference in the adult group (*p* = 0.036, Mann–Whitney). Adults with aortic dilatation showed lower OM diversity in terms of abundance (Figure 3B). A principal coordinate analysis (beta diversity, based on the Bray–Curtis index) showed the patients distribution with aortic dilatation and non-dilatation, with 3.8% of the variance related to the variables of this classification (R^2^ = 0.038, *p* = 0.64) (Figure 3C).

**Figure 4:**
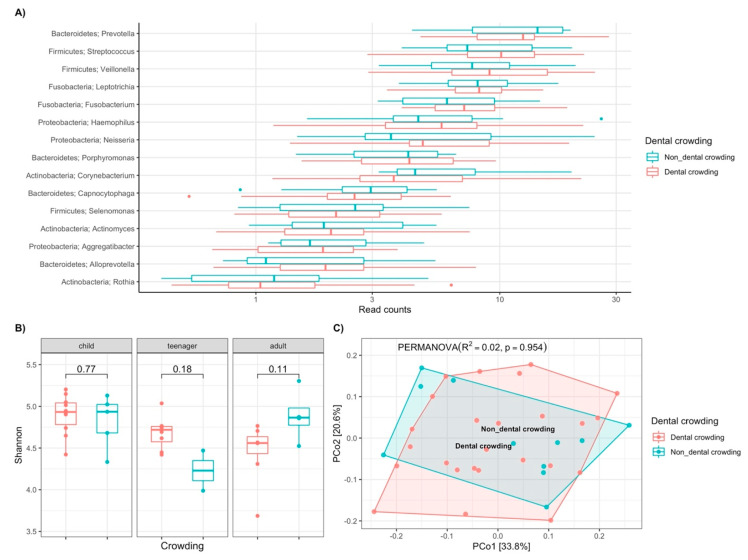
Analysis of oral microbiota in subjects with MFS according to dental crowding. **A**) Relative abundance of the main genera. **B**) Analysis of alpha diversity in relation to age groups and dental crowding based on the Shannon index. **C**) Principal coordinate analysis of beta diversity by Bray–Curtis index in relation to dental crowding (R^2^= 0,02, *p* = 0,954).

Subsequently, the previous analysis was performed using dental crowding as a grouping variable. Comparing the relative abundances of the 15 most abundant genera showed no significant differences between patients with and without dental crowding (Figure 4A).

When analyzing alpha diversity using the Shannon index across the three age groups, no significant differences were observed; however, in the teenager group with crowding, there was a higher abundance of genera (*p* = 0.18), while in the adult group with crowding, a lower abundance of genera was observed (*p* = 0.11) (Figure 4B). In the beta diversity analysis, no significant differences were found regarding the dental crowding variable, with only 2% of the variance related to this variable (R^2^ = 0.02, *p* = 0.954, PERMANOVA) (Figure 4C).

### 3.3. Cytokine Profile

The serum cytokine concentrations of MFS patients were measured. Upon analyzing the concentrations, it was observed that the non-dilated group presented higher concentrations of IL-1β (165.09 pg/mL), IL-4 (127.34 pg/mL), IL-10 (34.31 pg/mL), and TGF-1 (476.9 pg/mL). In the dilated group, higher concentrations of IL-6 (207.83 pg/mL), IL-8 (65.02 pg/mL), TNF (21.87 pg/mL), and IFN-γ (146.54 pg/mL) were recorded.

However, no significant differences in cytokine levels were observed between the presence of aortic dilatation and non-dilatation. Nonetheless, IL-1β levels were higher in non-dilatation patients (165.09 pg/mL) than in those with dilatation (117.15 pg/mL), with a significance of *p* = 0.057.

**Figure 5:**
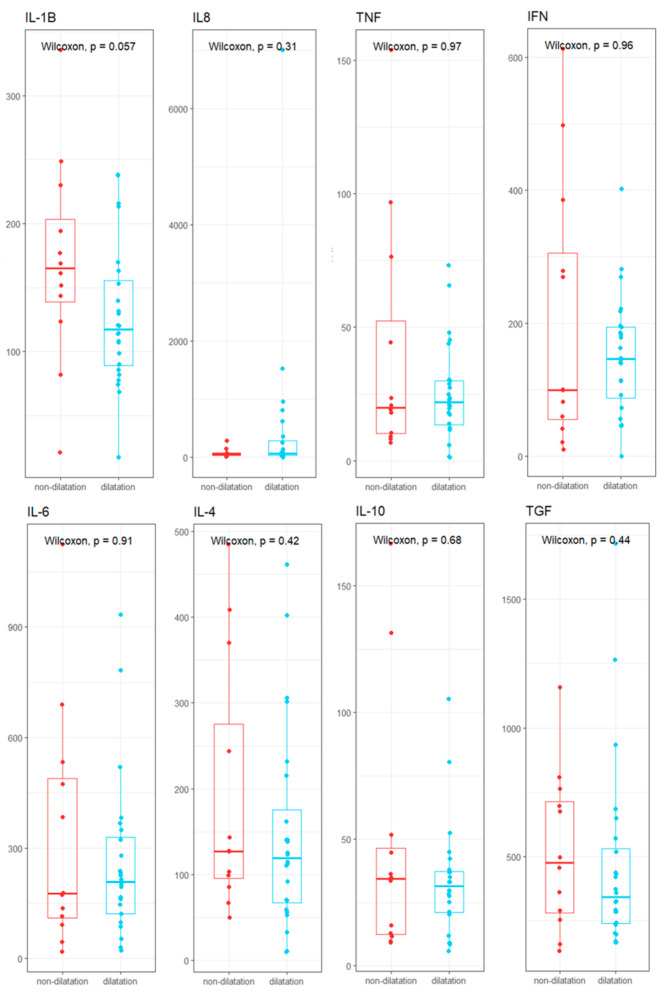
Serum cytokine concentrations (pg/mL) of MFS patients grouped by aortic dilatation/non-dilatation.

Statistical power analysis documented that a sample size of 22 patients would be required to detect significant differences (Figure 5, Supplementary Table S1).

### 3.4. Correlation Between Oral Microbiota and Cytokines

**Figure 6:**
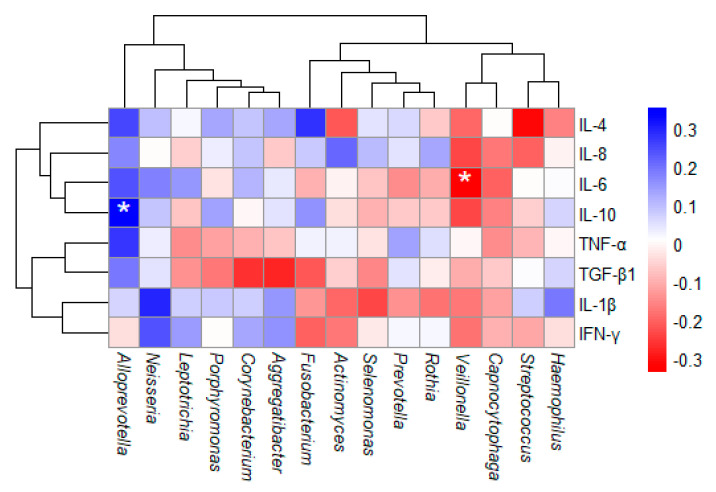
Spearman correlation test of oral microbiota diversity and serum cytokine concentrations in MFS patients. *p*-value ≤ 0.05. * Correspond to significant difference.

A Spearman correlation analysis between the 15 most abundant bacterial genera and cytokine levels was performed. A positive correlation was found between *Alloprevotella* and IL-10, while an anti-correlation was observed between *Veillonella* and IL-6 (Figure 6). No other significant associations were found in the analysis.

## 4. Discussion

This study provides the first description of the oral microbiota in patients with Marfan syndrome, showing that *Firmicutes* was the most abundant phylum in all three groups (children, teenagers, and adults), with its relative abundance increasing with age. *Bacteroidetes* was the second most abundant phylum, but unlike *Firmicutes*, its relative abundance was highest in children and decreased as age increased. It is well documented that the relative abundance of *Firmicutes* and *Bacteroidetes* varies greatly among individuals within the same population, which may be influenced by lifestyle factors, diet, hygiene habits, and other factors [25,26].

Regarding the genera, *Prevotella, Streptococcus*, and *Veillonella* were the most abundant in all three groups, without significant differences. However, the genus *Haemophilus* showed significant differences (*p* = 0.027), being more abundant in adults and teenagers. Species of the *Haemophilus* genus are part of the commensal microbiota but can occasionally switch to become opportunistic pathogens, infecting other parts of the body and causing potentially life-threatening conditions such as bacteremia, cellulitis, epiglottitis, meningitis, and pneumonia [27,28].

In the analysis of alpha diversity, significant differences were found between the child and teenager groups, with species richness and abundance decreasing with age. The child group showed the greatest diversity. Studies suggest that this increased diversity at an early age is related to factors such as birth method, diet, environmental influences, and genetics [29]. It has been described that age plays a critical role in MFS, as clinical manifestations and alterations tend to worsen as individuals reach adulthood. For example, the prevalence of cardiovascular anomalies in MFS is well known in adults but less understood in children and teenagers [30,31]. In our cohort, the number of major criteria for MFS diagnosis was not related to bacterial species abundance or richness. However, when analyzing the criterion of aortic dilatation, significant differences were found between adults with aortic dilatation and non-dilatation. Adults with aortic dilatation exhibited lower species abundance, with significant differences observed in three genera: *Fusobacterium* was less abundant in adults with dilatation, while *Actinomyces and Rothia* were more abundant in the same group.

*Fusobacterium* is a relevant component of the oral microbiome, playing a significant role in plaque formation, which makes it highly abundant in oral infections [32]. While *Fusobacterium* has been associated with cardiovascular diseases such as atherosclerosis and endothelial dysfunction, our study surprisingly documented that its abundance was lower in adults with aortic dilatation. Furthermore, not all *Fusobacterium* species have the same prevalence in the diseases to which they are linked. Therefore, it is crucial to update microbial detection technologies in clinical practice to ensure accurate disease diagnoses and identify individuals at risk [32,33].

The American Heart Association supports the association between periodontal disease and atherosclerotic vascular disease but does not currently support a causal relationship. Recently, it has been demonstrated that the main periodontal pathogens *Porphyromonas gingivalis* and *Treponema denticola* are causally associated with accelerated aortic atherosclerosis in ApoE-null hyperlipidemic mice.

However, there have been studies involving *Fusobacterium nucleatum* in experimental models that investigated whether this pathogen can accelerate aortic inflammation and atherosclerosis in the aortic artery of mice. Genomic DNA from *F. nucleatum* was detected in systemic organs (heart, aorta, liver, kidney, and lungs), suggesting bacteremia. The area of aortic atherosclerotic plaques was measured, and the local inflammatory infiltrate showed F4/80+ macrophages and CD3+ T cells. Vascular inflammation was detected by an increase in systemic cytokines (CD30L, IL-4, IL-12), oxidized LDL, and serum amyloid A, as well as by an altered serum lipid profile (cholesterol, triglycerides, chylomicrons, VLDL, LDL, and HDL) in infected mice and altered aortic gene expression in the same group. Despite the evidence of systemic infection in several organs and modulation of known atherosclerosis risk factors, aortic atherosclerotic lesions were significantly reduced after *F. nucleatum* infection, suggesting a potential protective role for this member of the oral microbiota.

Therefore, given our findings of lower levels of *Fusobacterium*, one potential explanation is that aortic dilatation would be expected to increase based on the theory presented earlier (R1A6).

A recent study reported the presence of *Rothia* in two patients with endocarditis, specifically in heart valve samples. This tissue is also commonly affected in MFS. Although the report mentioned that *Rothia* presence in this condition is rare (with only 23 reports worldwide), this may have been attributable to the difficulty of identifying this genus using conventional methods, with genomic sequencing being the most reliable identification technique [34]. Similarly, *Actinomyces* has been found in mitral valve tissue from patients with endocarditis and is also challenging to detect in blood cultures. One study mentions that 33 confirmed cases of *Actinomyces* in adult subjects were identified through genomic approaches [35].

While aortic dilatation is the leading cause of mortality in MFS, it is not the only cardiovascular issue. Mitral valve prolapse is an additional and significant alteration in these patients but it has received less attention. Reports show that mitral valve dysfunction is present in 80% of MFS patients, and by the age of 30, moderate to severe mitral insufficiency is found in one in eight individuals [36]. The identification of these bacterial genera in mitral valve tissue and the involvement of this tissue in MFS could suggest a link between the oral microbiome and cardiovascular alterations in MFS. However, this hypothesis requires further confirmation through more in-depth clinical and genomic studies.

FBN1 mutation directly affects the structure and functionality of the endothelial walls [37], leading to significant pathophysiological changes in the extracellular matrix, which impact receptors and cellular signaling pathways, as they are involved in the inflammatory state observed in these patients. In different conditions, when smooth muscle cells are dysfunctional, they can induce multiple disorders related to blood vessels, such as atherosclerotic coronary disease, aortic aneurysm, and aortic dissection [38], which are among the most severe complications in MFS.

In isolated vascular tissues from aortic dissection patients, the content of smooth muscle cells has been found to be diminished and associated with aortic dissection, suggesting that the aberrant function of smooth muscle cells within the aorta could be considered a significant cause of aortic dissection [39]. Their dysfunction in conditions of aortic dissection could result in the synthesis and release of inflammatory cytokines, including tumor necrosis factor-α (TNF-α), interleukin-1β (IL-1β), colony-stimulating factor (CSF), chemotactic factors, and growth factors (GFs). Substantial attention has been given to the potential roles of these cytokines in modulating the risk and pathological development of aortic dissection [40].

Although the statistical finding was borderline, we believe this was due to the small sample size, as a larger number of samples would be required to establish a clear difference in cytokines level. However, this exploratory study suggests that the coexistence of different mechanisms involving cytokines plays a role. To confirm this finding, further studies specifically focused on the action of these cytokines would be needed (R1A4).

Regarding IL-1β, it was found to have a lower concentration in patients with aortic dilatation, which aligns with a study in IL-1β-deficient mice. This study showed a decrease in aneurysm progression, suggesting that IL-1β could be a potential target for treating thoracic aortic aneurysms [41,42]. However, other factors may influence those data, such as age, sex, medication use, or other conditions that could affect IL-1β levels.

## 5. Conclusions

MFS is a poorly studied condition due to its rarity, the challenges associated with its diagnosis, and the limited available information, which results in few treatment options and strategies to improve the quality of life for these patients. We reported the first characterization of the oral microbiota in MFS patients. We acknowledge that this study has limitations due to the lack of additional clinical characteristics of each patient, which could enhance the understanding of the relationship between oral health and systemic conditions in MFS.

This preliminary exploration of oral microbiota in MFS opens up new perspectives for future translational studies, where oral and systemic health conditions and treatments could be evaluated through clinical trials, as well as studies focused on immune response mechanisms in the oral cavity and their potential connection to the local microbiome districts, including the gut.

In order to corroborate these results and enrich future studies, we plan to conduct trials aimed at clearly defining the relationship between the oral and intestinal microbiota in patients with Marfan syndrome. Additionally, we aim to gather clinical data specifically focused on each type of cardiovascular damage in these patients (R1, A7).

## Figures and Tables

**Table 1:** Demographic and clinical characteristics of patients with Marfan syndrome.

		Age groups			
	Total	Child	Adolescent	Adult	*p*-Value
**Patients**	36 (100.0)	15 (100.0)	10 (100.0)	11 (100.0)	
**Gender**					* 0.02883
Female	12 (33.3)	4 (27.7)	1 (10.0)	7 (63.6)	
Male	24 (66.7)	11 (73.3)	9 (90.0)	4 (36.4)	
**Age**					*** > 0.0001†
Range	5 – 55	5 - 13	14- 19	22 - 55	
	14.5 [11.0 – 24.25]	10 [6.5 – 12.0]	16 [14.3 – 16.8]	30 [26.0 – 36.0]	
	18.31 ± 11.38	9.46 ± 2.95	15.9 ± 1.73	32.55 ± 9.63	
**Major criteria (Ghent)**					*0.3038*
*Number of criteria*					
2	12 (33.3)	5 (33.3)	5 (50.0)	2 (18.2)	
3	10 (27.8)	2 (13.3)	3 (30.0)	5 (45.5)	
4	14 (38.9)	8 (53.3)	2 (20.0)	4 (36.4)	
*Systemic Score, pts*					*0.3527*
	7 – 14	7 - 12	7 - 14	7 -14	
	10 [7 – 12]	9 [7.0 – 12.0]	10 [9.3 – 12.0]	11 [9.0 – 12]	
	9.94 ± 2.34	9.27 ± 2.22	10.5 ± 2.51	10.64 ± 2.06	
*Aortic dilatation*	24 (66.7)	11 (73.3)	4 (40.0)	9 (81.8)	*0.105*
*Ectopia lentis*	23 (63.9)	10 (66.7)	6 (10.0)	7 (63.6)	*0.9452*
*Family history*	27 (75.0)	12 (80.0)	7 (70.0)	8 (72.7)	*0.838*
**Facial features (oral cavity)**					
*Deep palate*	29 (80.6)	13 (86.7)	7 (70.0)	9 (81.8)	*0.5915*
*Bifid uvula*	28 (77.8)	11 (73.3)	7 (70.0)	10 (90.9)	*0.4552*
*dental crowding*	25 (69.4)	10 (66.7)	8 (80.0)	7 (63.6)	*0.693*
**Caries risk,** **DMF-T index**					*** > 0.0001‡
Moderate	11 (30.6)			11 (100.0)	
Very high	25 (69.4)	15 (100.0)	10 (100.0)		

Notes: The data are reported as the number and percentage, n (%); range, min.–max. value; mean ± standard deviation; or median and (interquartile range). According to the tests of normality, homoscedasticity, and other features of the data distribution, a Kruskal–Wallis test was applied to analyze the difference between patients according to the age groups of MFS subjects. The two-tailed statistical significance at 95% was established at the following values: * *p* ≤ 0.05; *** *p* < 0.0001; † *p* = 2.023511 × 10^-7^; ‡ *p* = 2.511 × 10^−8^. DMF-T index scale in adults: very low: <1.2; low: 1.2–2.6; moderate: 2.7–4.4; high: 4.5–6.5; very high: > 6.5. Scale of the DMF-T index in infants: very low: < 5.0; low: 5.0–8.9; moderate: 9.0–13.9; high: ≥14.0.

## Data Availability

The datasets presented in this study are available in online repositories. Repository: SRA; access number: PRJNA869709.

## Supplementary Materials

The following information can be downloaded at https://www.mdpi.com/article/10.3390/biomedicines13020330/s1**. Table S1**: Cytokine levels (pg m L-1) of Marfan’s syndrome patients according to the presence or absence of aortic dilatation; the median is shown. *p* value, Wilcoxon test.
