# Supplementary material for: Differential Oral Microbiota and Serum Cytokine Signatures in Age-Grouped Patients with Marfan Syndrome
Source: Biomedicines. 2025 Jan 31;13(2):330. doi: 10.3390/biomedicines13020330 (PMC11853651; doi:10.3390/biomedicines13020330)
Supplement: Supplementary file 1 [file biomedicines-13-00330-s001.zip › biomedicines-3398767-supplementary.pdf]

Table S1. Cytokines' levels (pg m L^{-1}) of Marfan´s syndrome patients according to aortic dilatation and non-dilatation; showing the median. p value, Wilcoxon test.

	Cytokine´s concentrations [pg mL^{-1}] in serum sample			
	Cytokine type	Aortic non-dilatation (n=12)	Aortic dilatation (n=24)	*p-value*
IL-1β	Pro-inflammatory	165.09	117.1525	*0.057
TNF-α	Pro-inflammatory	19.93	21.87	0.97
IL-8	Pro-inflammatory	53.25	65.02	0.31
IL-6	Pro and anti-inflammatory	176.2	207.83	0.91
IFN-γ	Pro-inflammatory	99.6	146.5	0.96
IL-4	Anti-inflammatory	127.34	119.1	0.42
IL-10	Anti-inflammatory	34.31	31.4	0.68
TGF-β1	Anti-inflammatory	476.9	342.82	0.44
